# Supplementary material for: Development of a computer-aided design software for the quantitative evaluation of aesthetic damage
Source: PLoS One. 2019 Dec 18;14(12):e0226322. doi: 10.1371/journal.pone.0226322 (PMC6919621; doi:10.1371/journal.pone.0226322)
Supplement: S1 Code site — 1. (ZIP) [file pone.0226322.s001.zip › [EESC_jr][Projeto]Relatorio_07_21/reports/templates_zip/gentelella-master/vendors/morris.js/examples/updating.html]

# Updating data

```
var nReloads = 0;
function data(offset) {
  var ret = [];
  for (var x = 0; x <= 360; x += 10) {
    var v = (offset + x) % 360;
    ret.push({
      x: x,
      y: Math.sin(Math.PI * v / 180).toFixed(4),
      z: Math.cos(Math.PI * v / 180).toFixed(4)
    });
  }
  return ret;
}
var graph = Morris.Line({
    element: 'graph',
    data: data(0),
    xkey: 'x',
    ykeys: ['y', 'z'],
    labels: ['sin()', 'cos()'],
    parseTime: false,
    ymin: -1.0,
    ymax: 1.0,
    hideHover: true
});
function update() {
  nReloads++;
  graph.setData(data(5 * nReloads));
  $('#reloadStatus').text(nReloads + ' reloads');
}
setInterval(update, 100);
```
